# MRI surveillance after translabyrinthine vestibular schwannoma resection and cochlear implantation: is it feasible?

**DOI:** 10.1007/s00405-023-08036-3

**Published:** 2023-06-02

**Authors:** Valerie Dahm, Ursula Schwarz-Nemec, Michael A. Arnoldner, Rudolfs Liepins, Alice B. Auinger, Christian Matula, Christoph Arnoldner

**Affiliations:** 1 Department of Otorhinolaryngology, Head & Neck Surgery, Medical University of Vienna, Vienna, Austria; 2 Division of Neuroradiology and Musculoskeletal Radiology, Department of Biomedical Imaging and Image-Guided Therapy, Medical University of Vienna, Vienna, Austria; 3 Department of Neurosurgery, Medical University of Vienna, Vienna, Austria

**Keywords:** Cochlear implant, Vestibular schwannoma, Magnetic resonance imaging, Acoustic neuroma

## Abstract

**Purpose:**

Cochlear implantation in patients with vestibular schwannomas is of increasing importance and interest. Two remaining challenges are the assessment of conduction of the cochlear nerve and the possibility of postoperative surveillance with magnetic resonance imaging. The aim of the current study was to assess follow-up imaging and determine the visibility of the internal auditory canal after vestibular schwannoma resection and cochlear implantation as well as in patients with persistent vestibular schwannomas and cochlear implants in place. Visibility of the internal auditory canal, cerebellopontine angle, and labyrinth were evaluated and graded.

**Methods:**

For this retrospective study, 15 MR examinations of 13 patients after translabyrinthine vestibular schwannoma resection and ipsilateral cochlear implantation were included. All patients had been implanted with an MED-EL cochlear implant. Magnetic resonance imaging was carried out on a 1.5T device. All patients were prepped according to the manufacturer’s recommendations.

**Results:**

All 15 examinations were carried out without any adverse event during imaging, such as pain, magnet dislocation, or malfunction. The internal auditory canal and the cerebellopontine angle were sufficiently visible in all cases to allow for vestibular schwannoma follow-up.

**Conclusion:**

Magnetic resonance imaging surveillance of the internal auditory canal following vestibular schwannoma resection and cochlear implantation is feasible and safe with modern implants with a 1.5T magnetic resonance imaging device using metal artifact reduction sequences. Necessary follow-up imaging should not be a contraindication for cochlear implantation in patients with vestibular schwannomas.

## Introduction

Cochlear implantation in patients with vestibular schwannomas is of increasing importance and interest. Authors have called this treatment possibility with remarkable success a “game-changer” in the management of vestibular schwannomas [1].

Two remaining challenges are the assessment of conduction of the cochlear nerve and the possibility of postoperative surveillance with magnetic resonance imaging (MRI).

Manufacturers have addressed the issue of MRI compatibility over the past decades with the use of rotational magnets, for example. Although cochlear implants (CI) have been MRI compatible, several complications have been reported including malfunction, displacement, flipping of the magnet, heat and pain during imaging, and demagnetization [2]. Displacement, canting, or flipping of the magnet often leads to the need of revision surgery [3, 4]. In some cases, a manual manipulation through the scalp suffices to reposition the magnet [5, 6]. An additional important issue, especially in vestibular schwannoma patients, is the amount of artifact produced by the CI. The next step to improve MRI compatibility and reduce artifacts at the same time was the introduction of a rotating magnet (a diametrically bipolar magnet) [2, 7]. Rotating, self-aligning magnets have been shown to significantly reduce the amount of artifacts [8].

Further, studies have focused on positioning the implant at an ideal position for internal auditory canal (IAC) visibility, which seems to be at least 9 cm from the outer ear canal and at a more horizontal angle as well as more posterior [9, 10].

The removal of the magnet, which can lead to a period of anacusis, is another option. This, however, requires two surgical procedures for patients.

The aim of the current study was to assess follow-up imaging and determine the visibility of the IAC after vestibular schwannoma resection and cochlear implantation as well as in patients with persistent vestibular schwannomas and CI in place. Visibility of the IAC, cerebellopontine angle, and labyrinth were evaluated and graded.

## Materials and methods

The study protocol was approved by our institutional review board (EK 1486/2019) and the study procedure was performed in accordance with the Declaration of Helsinki. Due to the retrospective nature of this study, informed consent for study participation was waived.

### Study population

For this retrospective study, 15 MR (magnetic resonance) examinations of 13 patients after translabyrinthine vestibular schwannoma resection and ipsilateral cochlear implantation were included. Vestibular schwannoma was removed via a translabyrinthine approach and ipsilateral cochlear implantation was performed simultaneously. All patients were implanted with a MED-EL SYNCHRONY Implant (MED-EL Corporation, Innsbruck, Austria). Whether cochlear implantation was carried out after translabyrinthine tumor removal was decided after performing electrically evoked auditory brainstem response measurements with an intracochlear test electrode as described in the previous studies [11, 12]. The electrode array was 28 mm in six patients and 31.5 mm in seven patients. The distance between the cochlear internal magnet and the external auditory canal was 9–10 cm. Follow-up MR examinations were performed to rule out tumor recurrence. Eleven patients underwent one follow-up MR examination, and two patients had two MRI scans, respectively. MR examinations were performed at an average of 16 months after cochlear implantation. Demographic details are shown in Table 1.

**Table 1:** Demographic data of all 13 included patients with 15 MRIs, f—female, m—male, l—left, r—right

ID	Age (y)	Sex	Electrode	Time (CI-MRI)	Koos	Side	Examinations
1	45	m	Flex 28	11 m	2	r	Single
2	57	f	Standard	5 m	2	r	Multiple
3	60	f	Standard	34 m	2	r	Multiple
4	54	f	Standard	8 m	2	l	Multiple
5	56	f	Standard	34 m	2	l	Multiple
6	50	f	Flex 28	38 m	2	l	Single
7	48	f	Flex 31.5	13 m	IC	l	Single
8	45	m	Flex 31.5	11 m	2	l	Single
9	63	f	Flex 28	6 m	1	r	Single
10	56	f	Flex 31.5	3 m	2	r	Single
11	55	f	Flex 28	1 m	2	r	Single
12	60	f	Flex 31.5	6 m	2	r	Single
13	76	m	Standard	29 m	2	r	Single
14	46	f	Flex 28	5 m	2	l	Single
15	64	f	Flex 28	33 m	2	r	Single

The electrode column shows which MED-EL electrode was used. Time between cochlear implantation and magnetic resonance imaging is given in months

*Koos* Koos grade, *IC* intracochlear schwannoma. Eleven patients had one single examination; two patients had two MRIs (multiple)

### Imaging

The MR examinations of the cerebellopontine angle (CPA) were performed on a 1.5T (tesla) MRI unit (Magnetom, Vida, Siemens Healthineers AG, Erlangen, Germany) using a 20-channel head/neck coil. Prior to the examination, all external components of the cochlear implant were removed, and patients received a head wrap according to the recommendations of the manufacturer. Head wrap was carried out by an ENT specialist familiar with implant position and head wrap necessities. Surgical magnet removal was not carried out in any case.

The standardized MR imaging protocol included the following sequences relevant for this study: (a) a transverse turbo spin echo (TSE) T1-weighted (w) sequence with WARP metal artifact reduction with and without contrast media application [repetition time (TR), 500–600 ms; echo time (TE), 11 ms; flip angle 150°; field of view 256 × 256 mm; bandwidth, 540 Hz/Px; in-plane resolution, 0,9 × 0,9 mm; slice thickness, 2 mm; interslice gap 0,2 mm; view angle tilting, 80%]; (b) a coronal TSE T2-w sequence with WARP metal artifact reduction [repetition time (TR), 2500–3500 ms; echo time (TE), 80 ms; flip angle 150°; field of view 432 × 512 mm; bandwidth, 540 Hz/Px; in-plane resolution, 0,9 × 0,9 mm; slice thickness, 2 mm; interslice gap 0,2 mm; view angle tilting, 80%]; (c) a heavily T2-weighted three-dimensional sequence [constructive interference in steady state (CISS)] [repetition time (TR), 5,5 ms; echo time (TE), 2.8 ms; flip angle 80°; field of view 380 × 512 mm; bandwidth, 540 Hz/Px; in-plane resolution, 0.9 × 0.9 mm; slice thickness, 0.7 mm].

### Imaging evaluation

All examinations were anonymized and stored on a picture archiving and communication system (IMPAX, AGFA HealthCare) and randomly presented to a board-certified radiologist (U.S-N., 6 years of experience in head and neck imaging), who was not aware of any patient data.

A subjective visual grading system was used to assess image quality of three anatomical regions, namely the CPA, the IAC, and the labyrinth on the ipsilateral and contralateral side of the CI, respectively (Table 2). The labyrinth on the ipsilateral side of the CI only consisted of the cochlea due to the previous translabyrinthine surgery. It is important to note that this grading system is not validated. However, similar grading scales have been used in the previous studies [10, 13, 14].

**Table 2:** Detailed explanation of grading of image quality

Grade	Image quality	
Grade 0	Poor image quality	Significant artifacts, no visibility of anatomical structures, no diagnostic confidence
Grade 1	Fair image quality	Major artifacts, poor visibility of anatomical structures, poor diagnostic confidence
Grade 2	Good image quality	Insignificant artifacts, adequate visibility of anatomical structures, good diagnostic confidence
Grade 3	Excellent image quality	No artifacts, full visibility of anatomical structures, high diagnostic confidence

For the assessment of the interreader agreement, a board-certified radiologist (MAA, 5 years of experience in MRI) evaluated the same images in an independent reading session. For the assessment of the intrareader agreement, the images were re-evaluated by the board-certified radiologist 5 weeks after the first evaluation.

### Statistical analyses

All statistical analyses and graphs were performed using commercially available software (STATA 12.0, StatCorp, College Station, TX, USA). Cross-tabulation was used to demonstrate the absolute frequencies and percentages of visually graded anatomical regions. To assess differences of visibility of anatomical structures between the ipsilateral and contralateral side of the device, a visual grading characteristics analysis was performed. Weighted *K* statistics were used for the assessment of the interreader and intrareader agreement.

## Results

In total, 15 MRIs (13 patients) were included in the study. All patients underwent MRI without any adverse events, such as pain, heat, rotation of the magnet, or malfunction (Table 1). All CIs were fully functional after imaging was carried out.

**Fig. 1:**
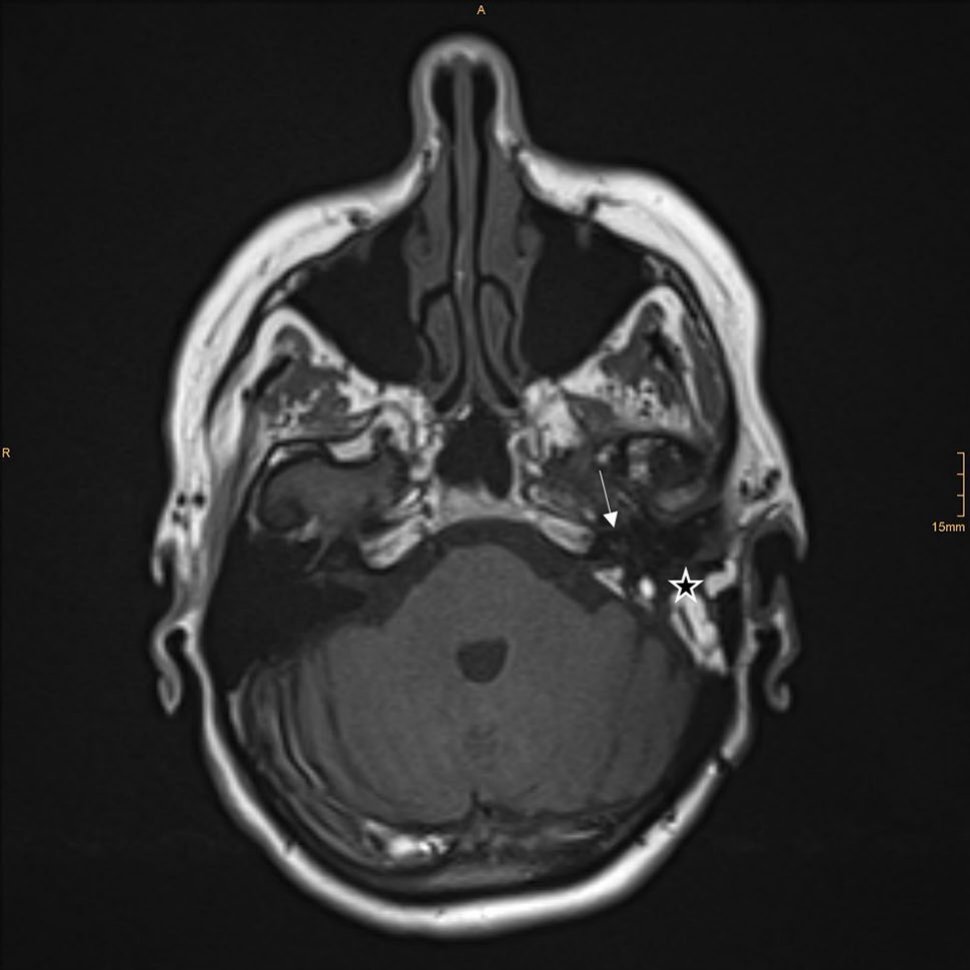
MRI T1, axial, postoperative image. The arrow points at the cochlea, the star is placed in the previous mastoid region showing postoperative changes. This image was rated as Grade 3—excellent image quality

**Fig. 2:**
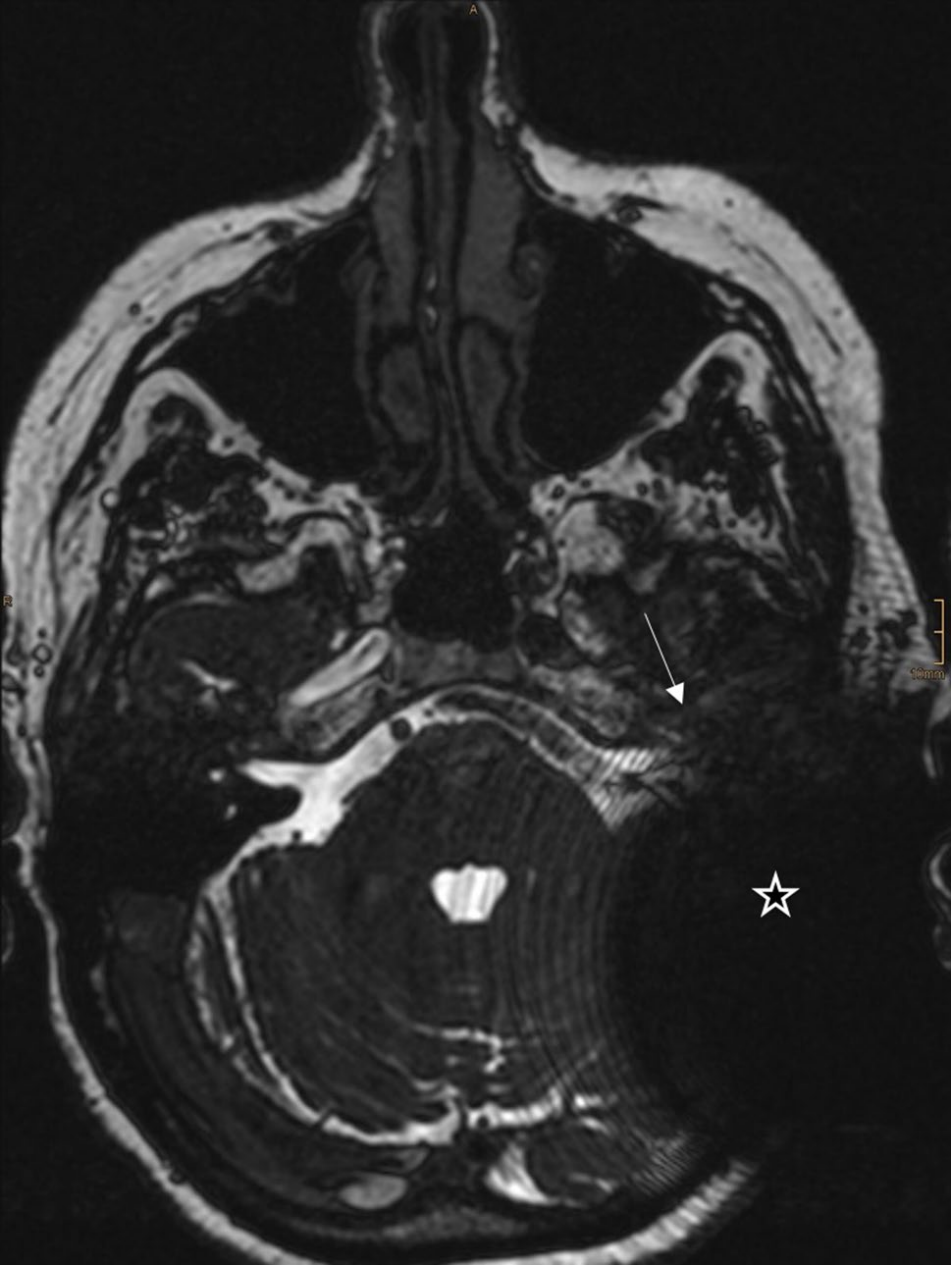
MRI CISS, axial, postoperative image. The arrow points at the cochlea (hardly visible), the star is placed in the large artifact created by the cochlea implant. This image was rated as Grade 0—poor image quality

**Fig. 3:**
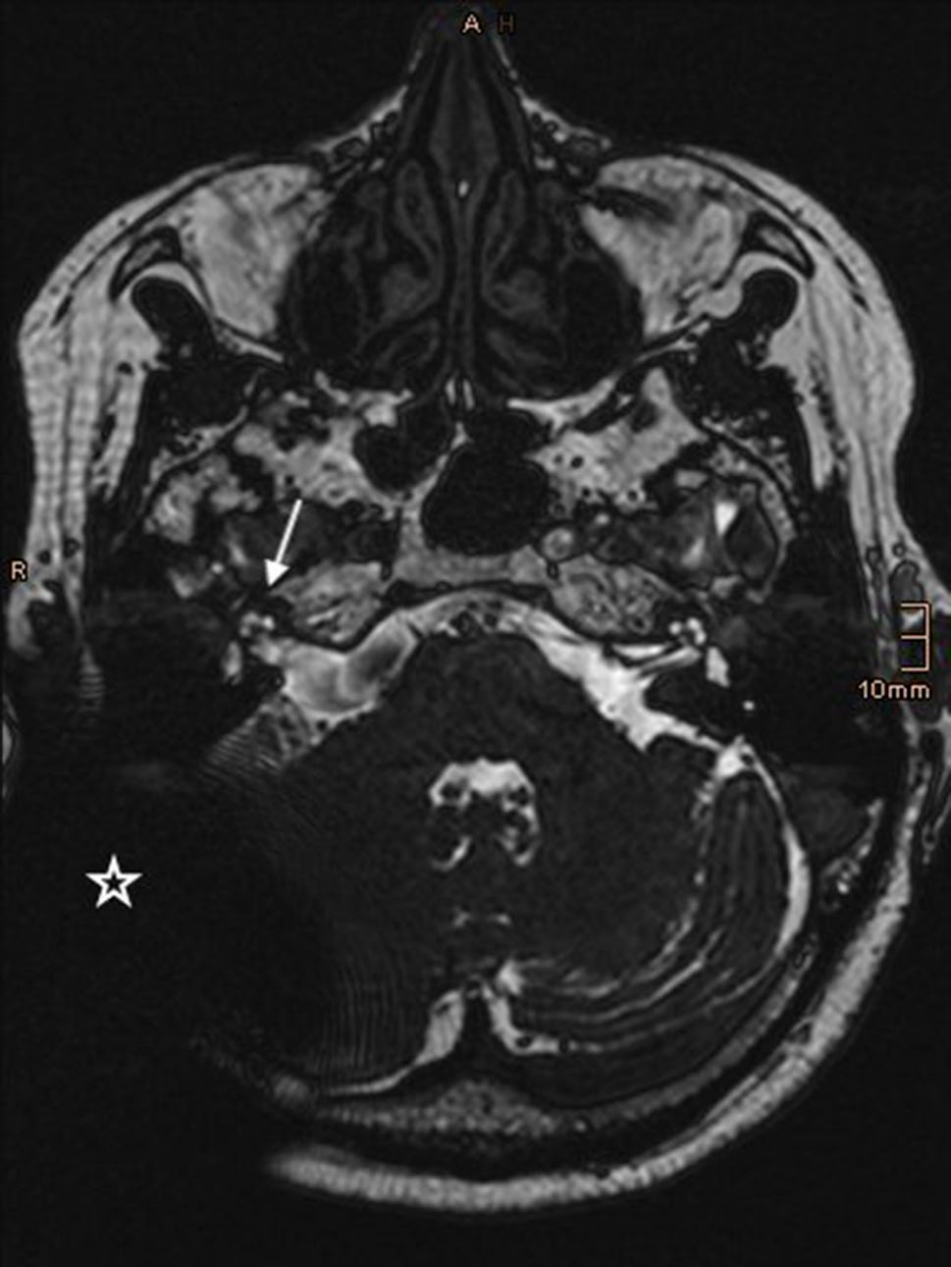
MRI CISS, axial, postoperative image. The arrow points at the cochlea, the star is placed in the large artifact created by the cochlea implant. This image was rated as Grade 2—good image quality

**Fig. 4:**
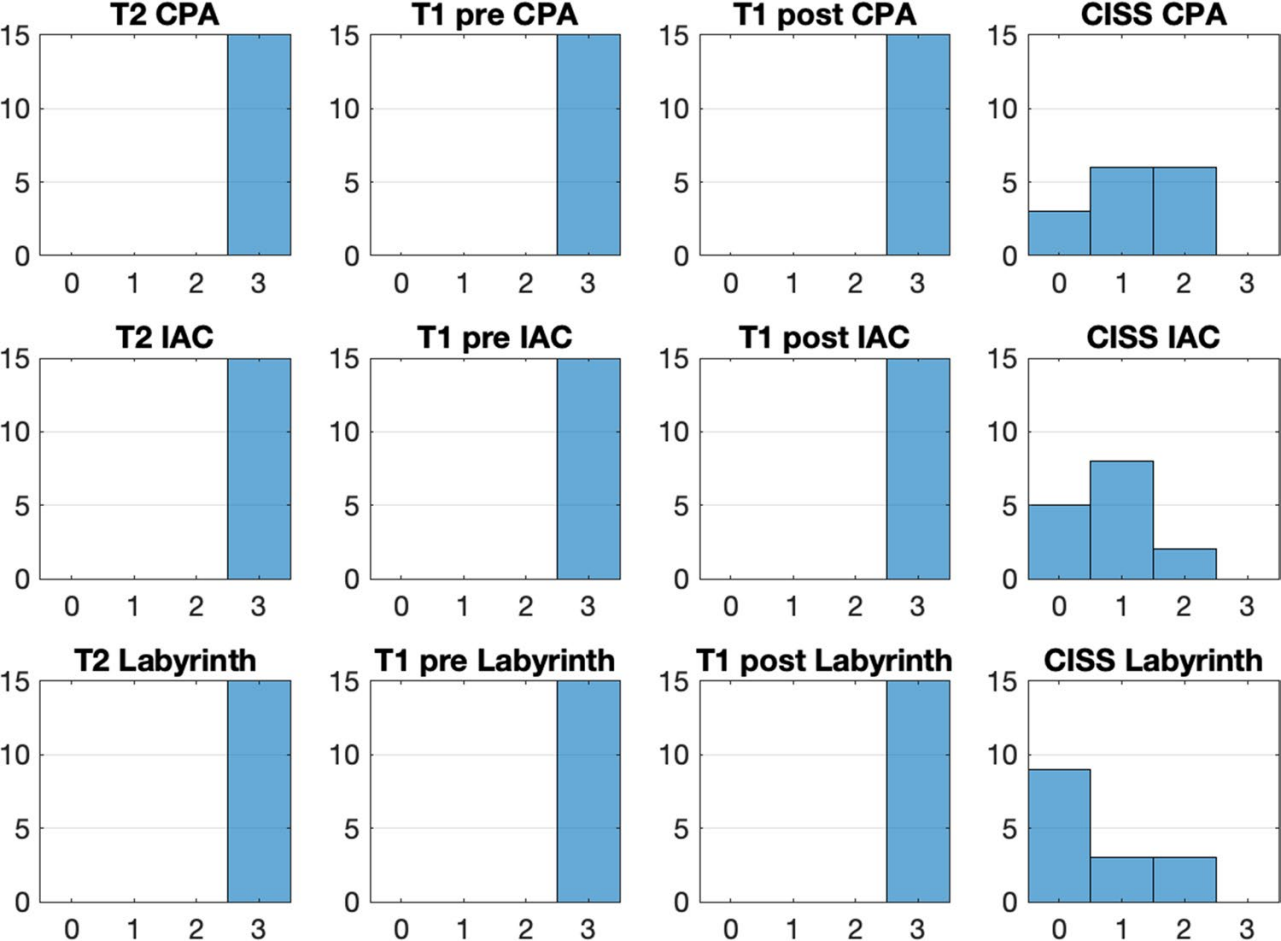
Imaging quality of ipsilateral structures. *CPA* cerebellopontine angle, *IAC* internal auditory canal, *T1 pre* before contrast agent, *T1 post* after contrast agent. *CISS* constructive interference in steady state. *Grade 0* poor image quality, *Grade 1* fair image quality, *Grade 2* good image quality, *Grade 3* excellent image quality

Artifacts and image quality were graded from 0 to 3 (0—poor image quality; 3—excellent image quality, see Table 2). The ipsi- and contralateral IAC, CPA, and labyrinth were evaluated. Results on the ipsilateral side show grade 3 (excellent image quality) of the CPA, IAC, and labyrinth (cochlea) on T2 and T1 pre- and post-images. Image quality on CISS images was between 0 and 2 (poor, fair, or good image quality). Only two patients had good image quality on CISS sequencing when examining the IAC, CPA, and labyrinth. Ten of the 15 included patients had at least one grade 0 on CISS sequencing for either the CPA, IAC, or labyrinth. Imaging examples are shown in Figs. 1, 2 and 3. Results of the imaging quality of ipsilateral structures are shown in Fig. 4.

Results on the contralateral side show grade 3 image quality on T2, T1 and CISS sequences when examining the CPA, IAC, and labyrinth. On the contralateral side, all relevant structures were therefore visible with excellent image quality (grade 3).

Interreader reliability was very high for T2 and T1 images (Kappa = 1). For CISS sequences, kappa was 0.82, 0.89, and 0.90 for the ipsilateral CPA, IAC, and labyrinth, respectively. The contralateral CISS images again showed excellent interreader reliability with a kappa of 1. Intrareader reliability showed a kappa of 1 in all sequences.

## Discussion

In this study, we present follow-up MRI results of patients who had undergone translabyrinthine vestibular schwannoma resection and cochlear implantation. The study shows that the IAC, CPA, and labyrinth are visible with excellent image quality on MRI (T2, T1 TSE imaging) using metal artifact reduction on the ipsilateral as well as on the contralateral side of patients with a CI in place. T1 TSE pre- and post-contrast agent, as well as T2 TSE MRI images were of sufficient quality to rule out residual or recurrent disease within the IAC, labyrinth, or CPA. CISS images were of poor, fair, or good quality. On heavily T2-weighted 3D sequences, the ipsilateral CPA, IAC, and labyrinth were not sufficiently visible in all patients. Heavily T2-weighted 3D sequences are mainly used to visualize the nerves within the IAC and to assess fibrosis of the cochlea in this context. Both these details are of little consequence after schwannoma excision and cochlear implantation. Although, high-resolution T2-weighted 3D sequences have been described to be sufficient in the assessment of recurrent tumor, T1-weighted pre- and post-contrast images are still the gold standard for postoperative imaging [15].

Additionally, to the assessment of residual or recurrent disease, cochlear implant malfunction or discomfort can be an issue during MRI. During our study, there were no incidences of pain, heat, or malfunction of the device in this group implanted with an MRI compatible CI with a rotating magnet.

Recently, Shew et al. published a study on MRI complications with CI in place. They reported on 15 CI patients, who underwent 24 MRI scans, of which five had complications. None of the five had an implant with a rotating magnet. The authors concluded that CI-MRI-related adverse events were occurring at an unacceptable frequency during their observation period [3]. They further wish to raise awareness that diagnostic MRI benefits must outweigh possible complications and advocate for continued industry technological innovation [3].

We agree that diagnostic imaging should be assessed for necessity, especially in face of possible complications. However, we believe that undergoing an MRI in an experienced center with a preparatory head wrap, and the use of MRI compatible CI's is safe.

One of the main arguments against cochlear implantation in patients with vestibular schwannomas can be that MRI follow-up is not possible. Our results show that follow-up imaging is not a contraindication for CI in this special patient group. Several further studies have reported on MRIs in patients with rotating magnets. Todt et al. reported on five implantees [9], as well as a further study by the same group reporting on six CI patients [16], Cass et al. presented one single patient [2], and Shew et al. reported on six patients among other individuals with CIs without rotational magnets [3]. None of these studies reported any incidences of pain, discomfort, or magnet dislocation. This is in line with the results of our study. The presented study is the largest study, so far, reporting on patient outcomes as well as artifacts with these implants. Studies have assessed artifacts and IAC visibility in cadavers as well as in patients with a CI [6, 8, 10, 16]. Only a few results have been published on patients after vestibular schwannoma resection and cochlear implantation undergoing follow-up imaging.

Schwartz et al. reported on six patients who underwent resection of unilateral sporadic vestibular schwannoma and cochlear implantation with an implant with a rotating magnet [13]. These six patients underwent eight follow-up MRIs. Ipsilateral IAC and CPA were partially or fully visualized in seven of the eight imaging studies. One implant was positioned in an atypical position, which was not further described in said study. Authors do encourage to position the implant at an exaggerated superior and posterior position, which has been advocated for by other authors as well [10, 17]. These recommendations were also followed in our patients as mentioned in the Materials and methods section.

Limitations of the current study include the retrospective character as well as the small sample size. Further, only patients with a CI of a single manufacturer were included. A prospective study with multiple manufacturers and blinded evaluation of MRI images is needed to draw further and more accurate conclusions. Nevertheless, these results show MRI safety and feasibility when following suitable protocols, using artifact reduction sequences, and following the manufacturer’s recommendations in specific devices.

## Conclusions

The presented study is the largest study to date evaluating IAC, CPA, and labyrinth visibility after translabyrinthine vestibular schwannoma resection and cochlear implantation. There were no cases of pain, device failure, or malfunction in the presented study. T1 and T2 TSE sequences were sufficiently interpretable to comment on tumor recurrence. Necessary follow-up imaging should, therefore, not be a contraindication for cochlear implantation in these patients.

## Data Availability

Data will be made available upon reasonable request.
